# Associated risk factors of urinary tract infection among pregnant women at Felege Hiwot Referral Hospital, Bahir Dar, North West Ethiopia

**DOI:** 10.1186/1756-0500-6-292

**Published:** 2013-07-25

**Authors:** Tazebew Emiru, Getenet Beyene, Wondewosen Tsegaye, Silabat Melaku

**Affiliations:** 1 Department of Microbiology, Immunology and Parasitology, College of Medicine and Health Sciences, Bahir Dar University, P.O.Box 79, Bahir Dar, Ethiopia; 2 Department of Laboratory Sciences and Pathology, College of Public Health and Medical Sciences, Jimma University, P.O Box 378, Jimma, Ethiopia; 3 Regional Health Research Laboratory, Bahir Dar, Northwest Ethiopia, Ethiopia

**Keywords:** Associated risk factors, Urinary tract infection, Pregnant women

## Abstract

**Background:**

Urinary tract infections (UTIs) are among the most common bacterial infections in humans, both in the community and hospital settings. It is a serious health problem affecting millions of people each year and is the leading cause of Gram-negative bacteremia. We previously conducted a study on “Urinary Bacterial Profile and Antibiotic Susceptibility Pattern of UTI among Pregnant Women in North West Ethiopia” but the study did not address risk factors associated with urinary tract infection so the aim of the study was to assess associated risk factors of UTI among pregnant women in Felege Hiwot Referral Hospital, Bahir Dar, North West Ethiopia.

**Methods:**

A total of 367 pregnant women with and without symptoms of urinary tract infection(UTI) were included as a study subject from January 2011 to April 2011. Midstream urine samples were collected and processed following standard bacteriological tests. Data concerning associated risk factors were collected using structured questionnaires and were processed and analyzed using Statistical Package for Social Science (SPSS version 16).

**Result:**

Bivarait analysis of socio-demographic characteristics and associated risk factors of UTI showed that family income level (family monthly income level ≤ 500 birr($37.85); P = 0.006, OR = 5.581, CI = 1.658, 18.793 and 501–1000 birr ($37.93-$75.70), P = 0.039, OR = 3.429, CI = 1.065, 11.034), anaemia (P = 0.003, OR = 4.388, CI = 1.776, 10.839), sexual activity (P = 0.032, OR = 3.520, CI = 1.197,10.363) and past history of UTI (P = 0.000, OR = 3.397, CI = 1.672, 6.902) were found to be factors significantly associated with increase prevalence of UTI. In contrast multiparity, history of catheterization, genitourinary abnormality, maternal age, gestational age and educational status were not significantly associated with UTI among pregnant women.

**Conclusion:**

In this study UTI was high among pregnant women in the presence of associated risk factor such as anaemia, low income level, past history of UTI and sexual activity.

## Background

Urinary tract infection (UTI) is the single commonest bacterial infections of mankind, to help in alleviating the problem we previously conducted a study on UTI which addressed the bacterial profile and antibiotic susceptibility pattern of UTI [1,2] but there was a need to address associated risk factors of UTI which may play important role in the prevention and control of this serious health problem which affect millions of people each year and which also lead to Gram-negative bacteremia [1,3]. UTI is not only common but the range of clinical effect varies from asymptomatic bacteriurea (ABU) to acute pyelonephritis [4]. Women with ABU during pregnancy are more likely to deliver pre-mature or low-birth-weight infants and have a 20 to 30-fold increased risk of developing pyelonephritis during pregnancy compared with women without bacteriuria [5]. Untreated ABU can also leads to the development of cystitis in approximately 30% of cases. In addition acute pyelonephritis has been associated with anaemia [6]. ABU may also be associated with an increase in neonatal mortality and a source for Gram negative septicaemia [7]. In addition it also associated with pre-eclampsia and chronic renal disease that has been cited as significant adverse obstetric outcome and medical conditions [8].

Pregnancy is one of the factors which increase the risk of UTI partly due to the pressure of gravid uterus on the ureters causing stasis of urine flow and is also attributed to the humoral and immunological changes during normal pregnancy [9]. During pregnancy there are a number of conditions associated with an increased prevalence of UTI [10]. UTI is common with varying prevalence by age, sexual activity and the presence of genitourinary abnormalities [5]. In healthy women, the prevalence of bacteriuria increases with age from about 1 percent in females with 5 to 14 years of age to more than 20 percent in women at least 80 years of age [5]. The prevalence is higher among individuals in lower socioeconomic classes and those with a past history of UTI [11]. Sickle cell traits, diabetes mellitus and grand multiparity have been reported; each is associated with two-fold increase in the rate of bacteriuria [10]. There is also increase in the risk of developing UTI due to catheterization, spermicidal contraceptive usage, kidney stones, tumors and urethral strictures [9,12]. Similarly increase risk of UTI reported in the presence of neurological diseases, congenital/acquired anomalies of bladder, vesico-ureteric reflux and suppressed immune system [9,12].

Though there are few studies [13-15] conducted on prevalence and antibiotic susceptibility pattern of UTI, there is no study conducted on associated risk factors of Urinary tract infection (UTI) in Ethiopia particularly in Bahir Dar town. Therefore, this study was designed to assess associated risk factors of UTI among pregnant women attended the antenatal care clinic of Felege Hiwot Referral Hospital, Bahir Dar, North West Ethiopia.

## Methods

The methods were previously described in our study on “Urinary Bacterial Profile and Antibiotic Susceptibility Pattern of UTI among Pregnant Women in North West Ethiopia” except few differences [1].

### Study site, study population, duration of study and data collection

A hospital based cross sectional study was conducted at Felege Hiwot Referral Hospital (FHRH) from January 2011 to April 2011 to determine associated risk factors of urinary tract infection (UTI) among pregnant women.

A pre-designed and structured questionnaire was used for the collection of data on associated risk factors. Collection of information on sign and symptoms of UTI and physical examination of pregnant women were done by Gynecologists and senior nurse.

Pregnant women who were taken antibiotics within seven days at the time of recruitment and who were not willing to participate were excluded from this study.

### Specimen collection and processing

Clean catch mid-stream urine samples were collected using sterile, wide mouthed glass bottles with screw cap tops. The pregnant women were also informed to clean their hands with water and then cleanse their periurethral area with sterile cotton swab soaked in normal saline to reduce the risk of contamination. Urine specimens were processed in the laboratory within 2 hours of collection and specimens that were not processed within 2 hours were kept refrigerated at 4°C until it was processed.

A calibrated sterile platinum wire loop was used for inoculation of specimens in to the culture media. It has a 4.0 mm diameter designed to deliver 0.01 ml. A loopful of well mixed urine sample was inoculated MacConkey, Manitol Salt Agar and Blood Agar (Oxoid, Ltd, England).

All plates were then incubated at 37°C aerobically for 24 hrs. The plates were then examined macroscopically for bacterial growth. The bacterial colonies were counted and multiplied by 100 to give an estimate of the number of bacteria present per milliliter of urine. A significant bacterial count was taken as any count equal to or in excess of 10,000 CFU/ml [3,7,8].

### Operational definitions

**Mid-stream urine specimen**: - a specimen obtained from the middle part of urine flow: Clean catch urine specimen.

**Symptomatic UTI** refers to patients whose urine is yielding positive cultures (≥ 10^5^CFU/ml) and who have symptoms referable to the urinary tract.

**Asymptomatic bacteriuria (ASB)** refers to the presence of two consecutive clear-voided urine specimens both yielding positive cultures (≥ 10^5^CFU/ml) of the same uropathogen, in a patient without urinary symptoms.

***Maternal Anaemia*** is defined as haemoglobin concentration less than 11 g/dl.

***Parity*** is the number of pregnancy reaching viability or beyond stage of abortion (before 20 weeks/less than 500 g BW).

***Gestational Age*** is the age of the fetus estimated by computing from the first day of the last menstrual period (time that precedes conception) until the day of consultation.

***History of UTI*** is any history of infection pertaining to the urinary tract diagnosed by a physician.

### Quality control

Pregnant women were informed to clean their hands with water and their genital area with swab soaked in normal saline before collection of the clean catch midstream urine samples. All specimens were transported from the hospital to regional laboratory within cold box and those specimens which were not processed with in 2hrs were kept in refrigerator and processed no longer than 18 hours after collection.

Only specimens which produced ≥ 10^5^CFU/ml of urine were considered significant but specimen who produced < 10^5^ colonies/ml of urine considered insignificant or due to contamination. Culture media were sterilized based on the manufactures instruction. Then the sterility of culture media were checked by incubating 3–5% of the batch at 35 – 37°C overnight and observed for bacterial growth. Those media which showed growth were discarded. The standard reference strains; *Staphylococcus aureus* (ATCC25923), *Escherichia coli* (ATCC25922) and *P. aeruginosa* (ATCC 27853) were used for testing quality of culture media.

### Ethical consideration

The study was conducted after getting a full approval by the health research unit of Jimma University, Amhara national regional state health bureau and FHRH. In addition written informed consent for the study was obtained from the study participants and confidentiality of results was kept. The results of urine tests were sent to the responsible person as soon as possible so that the pregnant women could be benefited from the study.

## Results

A total of 367 pregnant women (37 symptomatic and 330 asymptomatic pregnant women) were investigated for the presence of risk factors associated with urinary tract infections.

The assessment of associated risk factors of UTI showed that history of UTI (P = 0.000, OR = 3.397, CI = 1.672, 6.902), anaemia (P = 0.003, OR = 4.388, CI = 1.776, 10.839), sexual activity (P = 0.032, OR = 3.520, CI =  1.197,10.363) and family monthly income (taking family monthly income > 2000 birr ($151.40) as reference, family monthly income level ≤ 500 ETB ($37.85); P = 0.006, OR = 5.581, CI = 1.658, 18.793 and 501–1000 ETB($37.85-75.70); P = 0.039, OR = 3.429, CI = 1.065, 11.034) showed statistical significant association with UTI.

Bivariate analysis of other associated risk factors revealed that age of the pregnant women (taking age range 15–24 years as a reference, 25–34 years; P = 0.372, OR = 1.374, CI = 0.684, 2.763, 35–44 years; P = 0.999, OR = 0.000, CI = 0.000), gestational age (taking first trimester as a reference, second trimester; P = 0.251, OR = 0.558, CI = 0.206, 1.510, third trimester; P = 0.287, OR = 0.596, CI = 0.230,1.543), parity (taking nullipara as a reference: Multipara; P = 0.717, OR = 1.181, CI = 0.481, 2.901, Primipara; P = 0.134, OR = 0.536, CI = 0.237, 1.211), history of catheterization (P = 0.320,OR = 1.952, CI = 0.410, 9.286), genitourinary abnormality (P = 0.555, OR = 1.336, CI = 0.163, 11.432) and educational status (taking higher education as a reference, illiteracy; P = 0.361, OR = 1.701, CI = 0.544, 5.315, read and write; P = 0.233, OR = 2.867, CI = 0.509, 16.155) were not significantly associated with UTI (Table 1).

**Table 1:** Socio-demographic characteristics and associated risk factors of UTI among pregnant women at FHRH, Bahir Dar, January 2011- April 2011

	**Culture results**				
**Characteristics**	**Significant bacteriuria No (%)**	**No significant bacteriuria No (%)**	**Total No(%)**	**P, OR, ( 95% CI)**	
**Age**					
15-24	17 (8.5)	183 (91.5)	200 (54.5)		
25-34	18 (11.3)	141 (88.7)	159 (43.3)	0. 372, 1.374, (0.684, 2.763)	
35-44	0 (0)	8 (100)	8 (2.2)	0.999, (0.000)	
**Educational status**					
Illiterate	7 (10.6)	59 (89.4)	66 (18.0)	0.361, 1.701, (0.544, 5.315)	
Read and write	2 (16.7)	10 (83.3)	12 (3.3)	0.233, 2.867, (0.509, 16.155)	
Primary	7 (10.4)	60 (89.6)	67 (18.2)	0.376, 1.672, (0.535, 5.224)	
High school	13 (10.0)	117 (90.0)	130 (35.4)	0.365, 1.593, (0.582, 4.358)	
Higher education	6 (6.5)	86 (93.5)	92 (25.1)		
**Family monthly income (birr)**					
≤ 500 ($37.85)	10 (18.9)	43 (81.1)	53 (14.4)	0.006, 5.581, (1.658, 18.793)	
501-1000 ($37.93-$75.70)	12 (12.5)	84 (87.5)	96 (26.2)	0.039, 3.429, (1.065, 11.034)	
1001-1500 ($75.76-$113.55)	6 (9.5)	57 (90.5)	63 (17.2)	0.165, 2.526, (0.684, 9.334)	
1501-2000 ($113.63-$151.40)	3 (5.5)	52 (94.5)	55 (15.0)	0.678, 1.385, (0.298, 6.423)	
> 2000 ($151.40)	4 (4.0)	96 (96.0)	100 (27.2)		
**Gestational age**					
First trimester	7 (16.3)	42 (83.7)	49 (13.4)		
Second trimester	12 (8.5)	129 (91.5)	141 (38.4)	0.251, 0.558, (0.206, 1.510)	
Third trimester	16 (8.5)	161 (91.5)	177 (48.2)	0.287, 0.596, (0.230,1.543)	
**Noof sexual intercourses per week**					
Less than three	30 (8.7)	317 (91.3)	347 (94.5)	0.032, 3.520, (1.197,10.363)	
Three or more	5 (25.0)	15 (75.0)	20 (5.5)		
**Genitourinary abnormality**					
Yes	1 (12.5)	7 (87.5)	8 (2.2)	0.555, 1.366, (0.163, 11.432)	
No	34 (9.5)	325 (90.5)	359 (97.8)		
**History of catheterization**					
Yes	2 (16.7)	10 (83.3)	12 (3.3)	0.320,1.952, (0.410, 9.286)	
No	33 (9.3)	322 (90.7)	355 (96.7)		
**History of UTI**					
Yes	19 (18.1)	86 (81.9)	105 (28.6)	0.000, 3.397, (1.672, 6.902)	
No	16 (6.1)	246 (93.9)	262 (71.4)		
**Parity**					
Nullipara	17 (11.3)	133 (88.7)	150 (40.9)		
Primipara	10 (12.3)	146 (87.7)	156 (42.5)	0.134, 0.536, (0.237, 1.211)	
Multipara	8 (13.1)	53 (86.9)	61 (16.6)	0.717, 1.181, (0.481, 2.901)	
**Haemoglobin level**					
< 11 mg/dl	8 (27.6)	21 (72.4)	29 (7.9)	0.003, 4.388, (1.776, 10.839)	

## Discussion

Factors proposed to affect the frequency of bacteriuria during pregnancy include multiparity, gestational age, previous medical history of UTI, diabetes mellitus and anatomic urinary tract abnormalities [7,9,10,16-18]. In addition anaemia, socio-economic status, educational status, sexual activity and catheterization are also associated with increased risk of UTI [7,9,10,16-18].

To our knowledge this is the first study to assessed associated risk factors of UTI among pregnant women in the study area. In our study socioeconomic status, anaemia, sexual activity and history of UTI were shown to be the associated risk factors with UTI among pregnant women.

Our study findings showed that low socioeconomic status was one of the factors that were significantly associated with increased UTI. The frequency of UTI (18.9%) was higher among pregnant women who had family monthly income of less than 500 Ethiopian birr ($37.85) and 501–1000 Ethiopian birr ($37.93-$75.70) for the overall UTI. Similarly study on the same study subject in Pakistan by Haider *et al*[6] also showed that pregnant women who had low income level were more likely to have bacteriuria than those who had high socioeconomic income level. Another study in Egypt by Dimetry *et al*[19] on UTI also showed the presence of association between low income level and UTI. This could be due to the relation of low socioeconomic status with nutrition and immunity especially in pregnant women. On the contrary some studies reported the insignificant association between UTI and socio economic status [18,20].

Maternal anaemia was also significantly associated with UTI. Similar result were reported by different investigators such as study on UTI by Haider *et al*[6] in Pakistan, Taher *et al*[21] among asymptomatic pregnant women in Qatar, Enayat *et al*[10] among asymptomatic pregnant women in Iran and Sescon *et al*[7] among asymptomatic pregnant women in Philippines. This could be due to pregnant women with maternal anaemia are more likely to develop UTI than those who are not anaemic. The high probability of developing UTI among anaemic pregnant women may be related with immunity. A study on UTI by Kovavisarach *et al*[20] in Thailand reported the absence of association between anaemia and UTI.

The finding of this study also revealed that past history of UTI had strong association with UTI (P = 0.000, OR = 3.397, CI = 1.672, 6.902). Similar findings were reported by Haider *et al*[6] on UTI in Pakistan, Taher *et al*[21] in Qatar and Sescon *et al*[7] in Philippines among asymptomatic pregnant women. Masinde *et al*[22] also identified that past history of UTI is a risk factor for UTI during pregnancy. But absence of association was reported by Hamdan *et al*[23] in Sudan and by Kovavisarach *et al*[20] in Thailand.

Sexual activity was also the other associated risk factor that was found to be significantly associated with UTI. Pregnant women who had recent sexual intercourse of three or more per week were more likely to have UTI than women who had less than three intercourses per week. This may be due to sexual activity increases the chances of bacterial contamination of female urethra. Having intercourse may also cause UTIs in women because bacteria can be pushed into the urethra [3]. The anatomical relationship of the female urethra to the vagina makes it liable to trauma during sexual intercourse as well as bacteria can also be massaged up from the urethra into the bladder during pregnancy/child birth [3,24]. This finding is also in agreement with previous studies by Hooton *et al*[25] on UTI in Washington, Haider *et al*[6] on UTI in Pakistan and Amiri *et al*[17] on UTI among pregnant women in Iran.

Multiparity was associated with significant bacteriuria in pregnancy. This had been repeatedly recognized to cause a two-fold increase in the rate of ABU in pregnant women [8,26]. The association between multiparity and UTI is due to profound physiologic changes affecting the entire urinary tract during pregnancy has a significant impact on the natural history of UTI during gestation. These changes vary from patient to patient and are more likely to occur in women who have pregnancies in rapid succession [7]. According to this study parity was not significantly associated with UTI in pregnancy. This finding is in line with different studies throughout the world such as study by Hamdan *et al*[23] on UTI in Sudan, Masinde *et al*[22] on UTI in Tanzania, Turpin *et al*[11] among asymptomatic pregnant women in Ghana and Hazhir *et al*[27] among asymptomatic pregnant women in Iran. On the other hand other studies reported the presence of association between multiparity such as studies by Okonko *et al*[3] on UTI in Nigeria, Enayat *et al*[10] on asymptomatic pregnant women in Iran, Haider *et al*[6] on UTI in Pakistan.

There was no significant difference in the prevalence of UTI with respect to trimester. This is similar with earlier studies by Hamdan *et al*[23] on UTI in Sudan, Sheikh *et al*[18] on UTI in Pakistan, Masinde *et al*[22] on UTI in Tanzania, Hazhir among asymptomatic pregnant women [27] in Iran, Kovavisarach *et al*[20] on asymptomatic pregnant women in Thailand. Likewise level of education was also found to be not significantly associated with UTI. Similarly other studies also reported the absence of association between level of education and UTI among pregnant women such as study on UTI by Sheikh *et al*[18] in Pakistan, Masinde *et al*[22] on UTI in Tanzania, Hazhir [27] among asymptomatic pregnant women in Iran. There was no statistical significant association between age of the pregnant women and UTI. Similar finding also reported by Hamdan *et al*[23] on UTI in Sudan and Kovavisarach *et al*[21] on asymptomatic pregnant women in Thailand. The differences in results about the presence and absence of association between associated risk factors and UTI among different studies may be due to differences in methodologies, study population (living standard) and sample size used in the studies.

## Conclusions

In this study the chance of UTI was higher among pregnant women in the presence of associated risk factors such as anaemia, low income level, past history of UTI and sexual activity but there was no significant association between prevalence of UTI and risk factors such as multiparity, history of catheterization, genitourinary abnormality, maternal age, gestational age and educational status of pregnant women. Therefore pregnant women should be assessed for associated risk factors during their regular follow up.

### Availability of supporting data

There is no additional supporting data; the supporting data is included as Additional file 1.

## Competing interests

We verify that we all the authors have agreed to share the outcome of this manuscript equally and there is no conflict of interest among us.

## Authors’ contributions

DT was responsible for selection of the topic and write up of the proposal and also responsible for the final write up. BG was responsible for designing the methodology of the study and also involved in the selection of the topic whereas TW was responsible for the analysis and interpretation of the data. MS was responsible for collection of samples and data. All authors read and approved the final manuscript.

## Supplementary Material

Additional file 1 Questionnaire for socio-demographic, clinical data of symptomatic UTI and assessment of associated risk factors of UTI among pregnant women in FHRH. Click here for file
