# Supplementary material for: Associated risk factors of urinary tract infection among pregnant women at Felege Hiwot Referral Hospital, Bahir Dar, North West Ethiopia
Source: BMC Res Notes. 2013 Jul 25;6:292. doi: 10.1186/1756-0500-6-292 (PMC3750516; doi:10.1186/1756-0500-6-292)
Supplement: Additional file 1 — Questionnaire for socio-demographic, clinical data of symptomatic UTI and assessment of associated risk factors of UTI among pregnant women in FHRH. [file 1756-0500-6-292-S1.docx]

**Questionnaire for socio demographic, clinical data of symptomatic UTI and assessment of associated risk factors of UTI among pregnant women in FHRH**

**Date: ______________________**

**Code number: ____________________**

**Card number:____________________**

**Address**

**Zone_____________________________**

**Woreda___________________________**

**Kebele ___________________________**

**Tel number_______________________**

**Age _________________**

**I. Socio demographic characteristics**

1. Residence

Urban Rural

2. Educational level

> First degree First degree  Diploma High school Elementary school

Read and write  Illiterate

3. Marital status?

Married  Divorced  Widowed 

4. Occupation?

Governmental worker  Non-governmental worker 

Farmer  Merchant  student unemployed

5. Religion

Orthodox  Muslim protestant Catholic Other specify

6. Ethnic group

Amhara Oromo Tigray Other specify

7. Family monthly income?

<500 Birr  501-1000 Birr  1001-1500 Birr  1501-2000 Birr  > 2000 Birr 

**II. Clinical finding for symptomatic pregnant women**

1. Presence of burning sensation or pain during urination Yes  No

2. Presence of blood in the urine Yes No 

3. Frequency ( >7 urination per day and > 3 during the night time) Yes No

4. Urgency Yes No

5. Incomplete voiding Yes No

6. Presence of suprapubic pain Yes No

7. Presence of one or more of the following symptoms

Fever  flank pain  Rigors  nausea 

Vomiting  Loin pain or tenderness

**III. Risk factor assessment**

1. Presence of genitourinary abnormalities Yes No

2. Number of pregnancy---------------

3. History of abortion Yes No

4. Gestational age

First trimester Second trimester Third trimester

5. Haemoglobin level __________

6. History of urinary tract infection Yes No

7. Frequency of sex per week ----------------

8. History of use of catheterization Yes No
